# *Panax notoginseng* Flower Extract Attenuates Pentylenetetrazole-Induced Epilepsy by Restoring Glutamate Homeostasis

**DOI:** 10.3390/brainsci15101110

**Published:** 2025-10-15

**Authors:** Yang Zhao, Feiya Zhu, Jiayu Xie, Yiting Wang, Motlalepula Matsabisa, Minke Tang

**Affiliations:** 1 School of Chinese Materia Medica, Beijing University of Chinese Medicine, Beijing 102488, China; 2 African Medicines Innovations and Technology Development, School of Medicine, Faculty of Health Sciences, University of the Free State, Bloemfontein 9300, South Africa

**Keywords:** epilepsy, *Panax notoginseng* flower (PNF), neuroprotection, glutamate, neuroinflammation

## Abstract

**Objectives:** One-third of patients experience inadequate seizure control with antiseizure medications. Therefore, safer and more effective therapeutic strategies remain urgently needed. Research evidence indicates that *Panax notoginseng* flower may exhibit potential antiepileptic properties. The study aimed to investigate the neuroprotective and antiepileptic effects of *Panax notoginseng* flower (PNF) extract in a chronic pentylenetetrazole (PTZ)-kindled mouse model and explore its potential mechanisms, focusing on glutamate homeostasis. **Methods:** Chronic epilepsy was induced in ICR mice via repeated subconvulsive PTZ intraperitoneal injections. Following successful kindling, mice were orally treated with PNF (1.5 g/kg or 3 g/kg) for 30 days. Seizure behaviors were scored using Racines scale. Neuronal survival, systemic and cerebral cytokines, hippocampal glutamate levels (in vivo microdialysis with LC-MS/MS analysis), glutamate homeostasis related proteins glutamate transporter-2 (GLT-1), glutamate-aspartate transporter-1 (GLAST), and glutamine synthetase (GS) were investigated. **Results:** PNF treatment significantly reduced seizure severity and restored neuronal nuclei (NeuN+) cell neurons in the cortex and hippocampal CA1 region of PTZ kindled mice. PNF attenuated systemic and neuroinflammation by lowering interleukin-1ß (IL-1β), interleukin-6 (IL-6), and tumor necrosis factor-a (TNF-α) levels and increasing interleukin-10 (IL-10) in serum and brain of PTZ mice. PNF reduced hippocampal glutamate accumulation and upregulated GLT-1, GLAST, and GS expression, which were altered by PTZ stimulation. **Conclusions:** The PNF extract exhibits significant neuroprotective and antiepileptic effects in PTZ-kindled mice, likely through restoring glutamate homeostasis, and suppressing inflammation. These findings, with further clinical development, support the therapeutic potential of PNF as a complementary approach for epilepsy management.

## 1. Introduction

Epilepsy is one of the most common neurological disorders, affecting more than 70 million people worldwide, with over 75% of patients in low- and middle-income countries remaining untreated [1,2]. Clinically, approximately 20–30% of epilepsy patients are resistant to currently available antiepileptic drugs (AEDs) [3]. From a therapeutic perspective, the development of novel, safe, and effective antiepileptic agents remains an urgent unmet need.

The etiology of epilepsy is complex, and recurrent seizures can lead to neuronal damage. Patients often present with comorbid neurological disorders. Multiple hypotheses have been proposed to attempt to explain the pathophysiology of epileptic seizures, among which abnormal cortical hyperexcitability originating from epileptogenic foci is considered a key mechanism [4,5,6]. In the mammalian brain, the vast majority of excitatory neuronal activity is mediated by the neurotransmitter glutamate. Under physiological conditions, glutamate released into the synaptic cleft is rapidly cleared, with astrocytes responsible for the majority of glutamate uptake. Two major glutamate transporters have been identified on astrocytic membranes: glutamate transporter-1, also known as excitatory amino acid transporter 2, EAAT2 (GLT-1) and glutamate-aspartate transporter-1 (GLAST) [7]. Glutamate taken up by astrocytes can be converted to glutamine via glutamine synthetase and subsequently shuttled back to neurons, completing the glutamate-glutamine (Glu-Gln) cycle to maintain neuronal glutamate homeostasis [8]. Dysfunction of astrocytic glutamate uptake can lead to excessive synaptic glutamate accumulation, excitotoxicity, and ultimately neuronal injury or loss. Therefore, modulating astrocytic glutamate homeostasis represents a critical strategy to reduce hyperexcitability in the central nervous system.

In recent years, the therapeutic potential of traditional Chinese medicine (TCM) in neurological disorders has attracted increasing attention. Our previous studies investigated the antiepileptic effects of ginsenosides, particularly ginsenoside Rb1, and demonstrated that Rb1 significantly ameliorates experimental seizures [9,10]. Further mechanistic studies suggest that its effects may be mediated via G protein-coupled receptor-stimulating G protein (GPCR-Gs) signaling [11]. Building upon this work, we explored medicinal plants containing ginsenoside Rb1 and discovered that *Panax notoginseng* flower exhibits a distinct saponin profile compared with traditional *Panax ginseng* and including *Panax notoginseng* roots. *Panax notoginseng* flowers, which are widely used as herbal tea in China, have the highest content of saponins compared to the other parts of *Panax notoginseng*. Notably, unlike the root, *Panax notoginseng* flower is rich in protopanaxadiol-type saponins, with a particularly high content of ginsenoside Rb1 [12,13,14]. However, whether *Panax notoginseng* flower possesses antiepileptic properties remains unknown.

Therefore, the primary aim of this study was to investigate the potential therapeutic effects of *Panax notoginseng* flower on in vivo experimental epilepsy. We established a chronic pentylenetetrazole (PTZ)-induced kindling model in mice. Following successful kindling, animals received treatment with *Panax notoginseng* flower extract (PNF) to evaluate its efficacy and to explore potential mechanisms. Our findings demonstrate that PNF significantly alleviates PTZ-induced experimental epilepsy, potentially through modulation of excitatory amino acid homeostasis.

## 2. Materials and Methods

### 2.1. Experimental Mouse Model

Male ICR mice (80 individuals, 20 per group, aged 6–8 weeks, 18–22 g) were purchased from SPF (Beijing) Biotechnology Co., Ltd. (Beijing, China). All animal experiments were conducted under the guidelines approved by the Experimental Animal Ethics Committee of Beijing University of Chinese Medicine (BUCM-1-2025022601-1008, 26 February 2025, License No. SCXK [Jing] 2004-0001). Mice were housed under controlled conditions (23 ± 1 °C, 30–70% humidity) with a 12 h light/dark cycle starting at 8:00 A.M., and had ad libitum access to food and water.

### 2.2. Drugs and Reagents

*Panax notoginseng* flower was obtained from Xin Juyi Guang Health Technology Co., Ltd. (Anguo, Baoding, China) and extracted with 50% ethanol water solvent (*v*/*v*). Ethanol was recovered by rotary evaporation at 40 °C under reduced pressure, and the extract was diluted with distilled water to 0.3 g crude drug/mL. The extract was then named PNF to indicate the extract of Panax notoginseng flower. Microdialysis probes (Molecular Weight Cut-Off:13kD, Spectra/Por^®^) were provided by Yisheng Changqing Biotechnology Co., Ltd. (Beijing, China). Pentylenetetrazole (PTZ, A20J10L91178) was purchased from Macklin Biotech (Shanghai, China). Sodium valproate (VPA, K1823102) was obtained from Aladdin (Shanghai, China). Sodium pentobarbital (PB, 020402) was supplied by the Institute of Chemical Reagents Co., Ltd. (Beijing, China).

### 2.3. Epileptic Kindling and Treatment

After 7 days of acclimatization, mice were randomly divided into control and epileptic kindling groups. The epileptic kindling group received intraperitoneal injections of subconvulsive PTZ (35 mg/kg) every 48 h until successful kindling, while the control group received an equivalent volume of saline [15]. Upon successful epileptic kindling, mice were randomly assigned to following groups: PTZ, PTZ + PNF3, and PTZ + PNF1.5, with 18 successful kindling mice in each group. Two mice were randomly removed from the control group, resulting in 18 mice in each group. After grouping, intragastric administration was initiated: the PTZ group received saline; the two PNF groups received 3.0 g/kg or 1.5 g/kg PNF, respectively, determined with reference to the customary daily consumption of Panax notoginseng flower in traditional Chinese practice. The control group also received saline. All treatments were administered once daily for 30 days. During this period, PTZ injections for the kindled mice were reduced from every 48 h to once every 5 days; the control group continued to receive saline injections.

### 2.4. Seizure Behavior Assessment

Immediately after PTZ injection, mice were placed in transparent observation boxes for 30 min, and seizure behaviors were scored according to a modified Racines scale [16]:

0—no abnormal behavior;

1—ear and facial twitching;

2—neck muscle jerks;

3—myoclonic jerks;

4—generalized clonic seizures;

5—generalized tonic–clonic seizures;

6—death.

Kindling was considered successful when mice displayed seizure scale of 4–5 on three consecutive occasions.

### 2.5. Brain Microdialysis

Mice were anesthetized with 0.5% sodium pentobarbital and securely positioned in a stereotaxic apparatus for probe implantation. Following scalp incision and skull exposure, a small burr hole with a diameter of 1.5 mm was drilled at the predetermined coordinates using a microdrill. A microdialysis probe was then stereotaxically implanted into the hippocampus and secured with glass ionomer cement. The mice were allowed to recover individually in cages before connecting to the microdialysis system. Artificial cerebrospinal fluid was perfused at 0.1 mL/h. Dialysates from the first hour were discarded, and the second-hour samples were collected and stored at −80 °C for subsequent analyses.

### 2.6. HPLC–MS/MS

Fifty microliters of dialysate were mixed with three volumes of 60% acetonitrile and kept at 4 °C for 30 min, followed by centrifugation at 10,000× *g* for 20 min at low temperature. Supernatants were vacuum-dried at 4 °C, reconstituted in 75% methanol (5×), and stored at −20 °C. The HPLC gradient was optimized as follows (mobile phase II: acetonitrile; mobile phase I: 0.1% formic acid in water): 0–12 min: 8%→30% I; 12–14 min: 30% B; 14–16 min: 30%→8% I; 16–19 min: 8% I. Injection volume was 5 µL. A standard curve method was used to quantify amino acids in each dialysate sample. A LC-MS system (UHPLC-Q-Exactive Orbitrap MS) from Thermo Fisher Scientific (Waltham, MA, USA) was used in this study.

### 2.7. Immunofluorescence

The brains of the mice were dissected to remove the cerebellum, then fixed in 4% paraformaldehyde, embedded in paraffin, and sectioned at a thickness of 4 µm. Primary antibodies: GLT-1 (ab205248, 1:1000, Abcam, Waltham, MA, USA); Neuronal Nuclei (NeuN 26975-1-AP, 1:100, ProteinTech, Rosemont, IL, USA), GLAST (20785-1-AP, 1:400, ProteinTech), and Glutamine synthetase (GS, 11037-2-AP,1:200, ProteinTech). Secondary antibody: Cy3 conjugated Goat Anti-Rabbit IgG (H + L) (1:300, GB21303, Servicebio, Wuhan, China). Sections were incubated with primary antibodies overnight at 4 °C and with secondary antibody at 23 ± 2 °C for 50 min. Nuclei were counterstained with DAPI in antifade medium. Images were acquired under a fluorescence microscope (NIKON ECLIPSE C1 with NIKON DSU3, Nikon Corporation, Tokyo, Japan), NeuN positive cells in cerebral cortex and hippocampus CA1 were counted using ImageJ1.

### 2.8. Measurement of Inflammatory Cytokines

Mice were anesthetized with 1% sodium pentobarbital (80 mg/kg), and 1 mL of blood was collected from the retro-orbital venous plexus. Serum was separated for cytokine measurement. Brains were dissected (cerebellum removed), homogenized, and supernatants were collected for tissue cytokine analysis. Tumor necrosis factor-alpha (TNF-α, KT2132-A), interleukin-1beta (IL-1β, KT2040-A), interleukin-6 (IL-6, KT2163-A), and interleukin-10 (IL-10, KT2176-A) (Changzhou, China), were measured using ELISA kits according to the manufacturer’s instructions. A standard curve based on five standard concentrations was fitted using linear regression, and the model was regarded as statistically reliable when the coefficient of determination (R^2^) was greater than 0.99.

### 2.9. Western Blotting

Brains (without cerebellum) were lysed using RIPA buffer (Coolaber, SL1020, Beijing, China). Protein concentrations were determined using a BCA kit. Proteins (40 µg) were separated by SDS-PAGE and transferred to PVDF membranes. Membranes were blocked with 5% non-fat milk in TBST for 2 h and incubated overnight at 4 °C with primary antibodies: GLT-1 (ab205248, 1:1000, Abcam), GLAST (20785-1-AP, 1:1000, ProteinTech), GS (11037-2-AP, 1:1000, ProteinTech), and β-tubulin (10094-1-AP, 1:2000, ProteinTech). Secondary antibody HRP (1:5000) was applied for 1 h at 4 °C. Bands were visualized using enhanced chemiluminescence, imaged on a Bio-Rad system (Hercules, CA, USA), and quantified using ImageJ software.

### 2.10. Statistical Analysis

The data were analyzed using SPSS 26. Homogeneity of variance was first assessed. Group differences were analyzed using one-way ANOVA. Significant ANOVA results were followed by pairwise comparisons using the least significant difference (LSD) post hoc test. A *p*-value < 0.05 was considered statistically significant.

## 3. Results

### 3.1. Effects of PNF on Seizure Behaviors in PTZ-Kindled Mice

**Figure 1:**
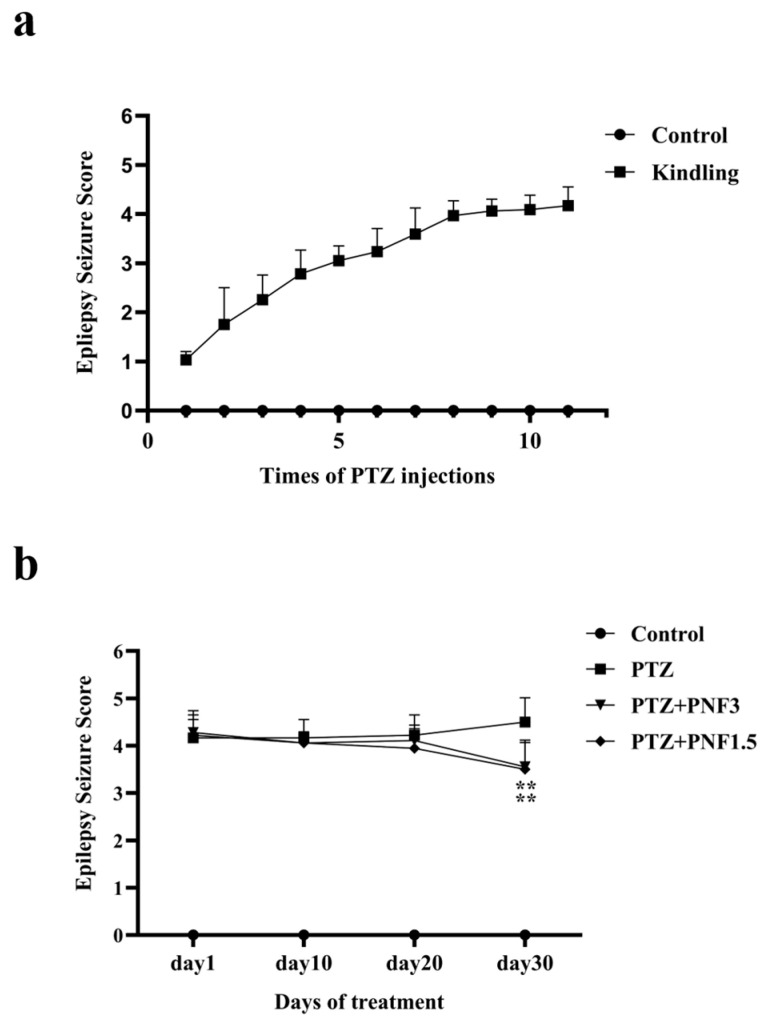
Effects of PNF on seizure behaviors in PTZ-kindled mice. The epileptic kindling in mice was induced with PTZ (35 mg/kg) by intraperitoneal injection (i.p.) every 48 h. Successful epileptic kindling was set as the mice with behavior scores of 4 to 5 according to Racines methods. The corresponding scores are as follows: 1 for ear and facial twitching, 2 for neck muscle jerks; 3 for myoclonic jerks; 4 for generalized clonic seizures; 5 for generalized tonic–clonic seizures; 6 for death. PTZ-kindled mice were treated with PNF 3.0 g/kg or 1.5 g/kg by intragastric (i.g.) once daily consecutively for 30 days. During the treatment period, PTZ injections for the kindled mice were reduced from every 48 h to once every 5 days. (**a**) The epileptic scores during kindling with PTZ; (**b**) PTZ-kindled mice treated with PNF. Data are presented as mean ± SEM (*n* = 18); ** *p* < 0.01 vs. PTZ group.

During Epileptic Kindling, mice in the kindling group progressively developed facial twitching, tail flicking, and bilateral forelimb jerks, followed by tonic extension of the hind limbs or generalized clonic-tonic seizures with falls. These symptoms became more pronounced with prolonged PTZ administration. After the 11th intraperitoneal injection of PTZ, according to a modified Racines scale [16], seizure scores of the epileptic kindling mice reached 4 or 5 (Figure 1a), indicating successful establishment of the PTZ kindling model. In contrast, no atypical behavior was observed in the control group.

Following successful epileptic kindling, the PTZ-kindling mice were regrouped, and treatment was initiated. As the treatment progresses, seizure scores in PNF-treated mice gradually decreased from day 10 onward. After 30 days of treatment, tonic hind-limb extension and generalized convulsions were markedly alleviated in PNF treated mice. Seizure scores in all PNF-treated groups were significantly lower than those in the untreated PTZ group (Figure 1b) (*p* < 0.01).

### 3.2. Effects of PNF on Cortical and Hippocampal Neurons in PTZ-Kindled Mice

Prolonged seizures can lead to neuronal damage, which in turn increases the complexity and severity of epilepsy. For these reasons, in order to label neurons and evaluate neuronal changes in the cerebral cortex and hippocampus in the different experimental groups, NeuN immunostaining was used. During the treatment phase, PTZ-induced kindling stimulation was maintained to evaluate the differences between treated and untreated groups.

**Figure 2:**
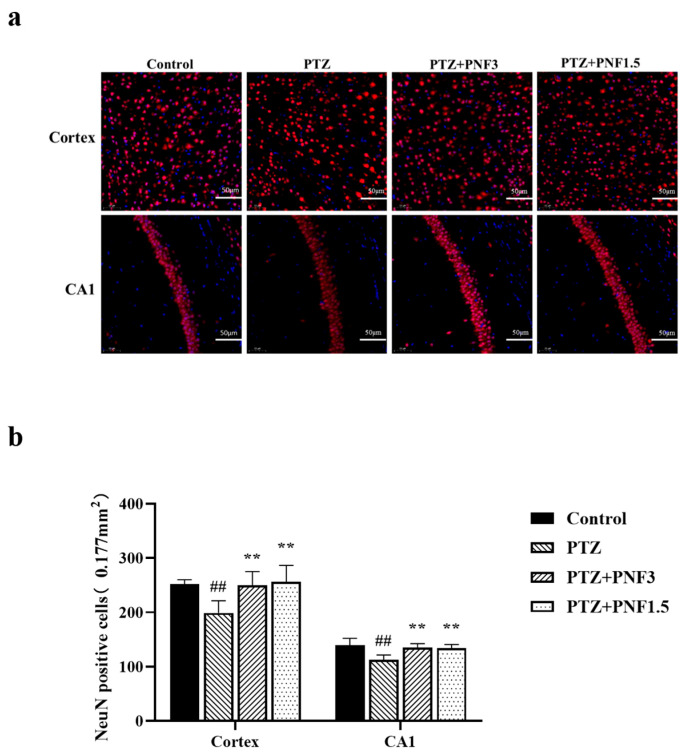
PNF preserves cortical and hippocampal neurons in PTZ-kindled mice. (**a**) Representative images of NeuN immunofluorescence staining in the cerebral cortex and hippocampal CA1 region across experimental groups. Scale bar = 50 µm; (**b**) NeuN positive cells in cerebral cortex and hippocampus CA1 were counted using ImageJ. The results show a significant neuronal loss in the PTZ group, which was alleviated by PNF treatment at both doses. Data are presented as mean ± SEM (*n* = 6); ^##^ *p* < 0.01 vs. control group; ** *p* < 0.01 vs. PTZ group.

In the untreated PTZ group, the number of NeuN^+^ neurons in the hippocampal CA1 region and cerebral cortex was markedly reduced (Figure 2a). In contrast, both 3.0 g/kg and 1.5 g/kg PNF treatment significantly preserved NeuN^+^ neurons in these regions, with cell counts being notably higher than those in the untreated PTZ group (Figure 2b) (*p* < 0.01).

### 3.3. Effects of PNF on Serum Inflammatory Cytokines in PTZ-Kindled Mice

**Figure 3:**
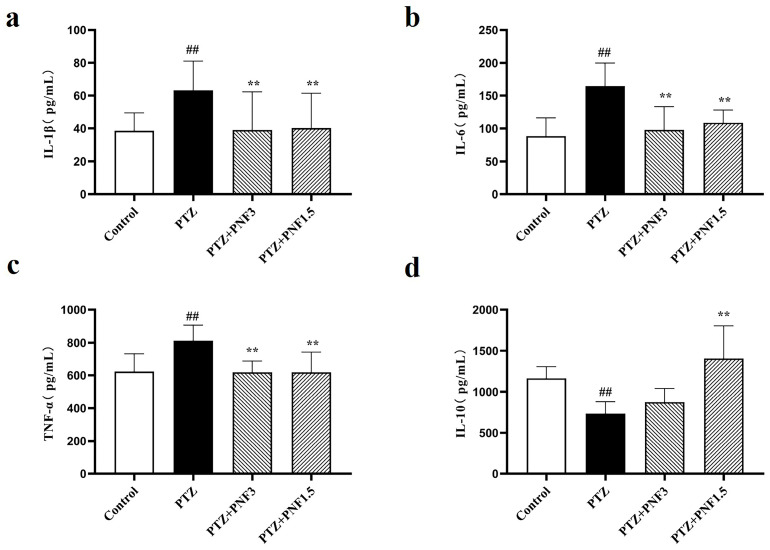
Effects of PNF on serum inflammatory cytokines in PTZ-kindled mice. (**a**) Levels of pro-inflammatory cytokines IL-1β; (**b**) Levels of pro-inflammatory cytokines IL-6; (**c**) Levels of pro-inflammatory cytokines TNF-α; (**d**) Levels of anti-inflammatory cytokines IL-10. PNF treatment (1.5 g/kg and 3.0 g/kg) significantly reduced pro-inflammatory cytokines and restored IL-10 levels. The cytokines were detected with ELISA. A standard curve based on five standard concentrations was fitted using linear regression, and the model was regarded as statistically reliable when the coefficient of determination (R^2^) was greater than 0.99. Data are presented as mean ± SEM (*n* = 8); ^##^ *p* < 0.01 vs. control group; ** *p* < 0.01 vs. PTZ group.

To monitor the systemic inflammation, after 30 days of treatment, specific cytokines in the serum were detected with ELISA. The results showed that, compared with the control group, mice in the PTZ group exhibited significantly elevated serum levels of the pro-inflammatory cytokines IL-1β (Figure 3a), IL-6 (Figure 3b), and TNF-α (Figure 3c) (*p* < 0.01), accompanied by a marked decrease in the anti-inflammatory cytokine IL-10 (Figure 3d) (*p* < 0.01), indicating that chronic PTZ kindling induced a systemic inflammatory response in mice.

Treatment with PNF at both 1.5 g/kg and 3.0 g/kg significantly reduced serum IL-1β (Figure 3a), IL-6 (Figure 3b), and TNF-α levels (Figure 3c) (*p* < 0.01) and increased IL-10 (Figure 3d) levels compared with the untreated PTZ group. These results suggest that PNF intervention effectively attenuated PTZ-induced systemic inflammation in mice.

### 3.4. Effects of PNF on Inflammatory Cytokines in the Brain of PTZ-Kindled Mice

**Figure 4:**
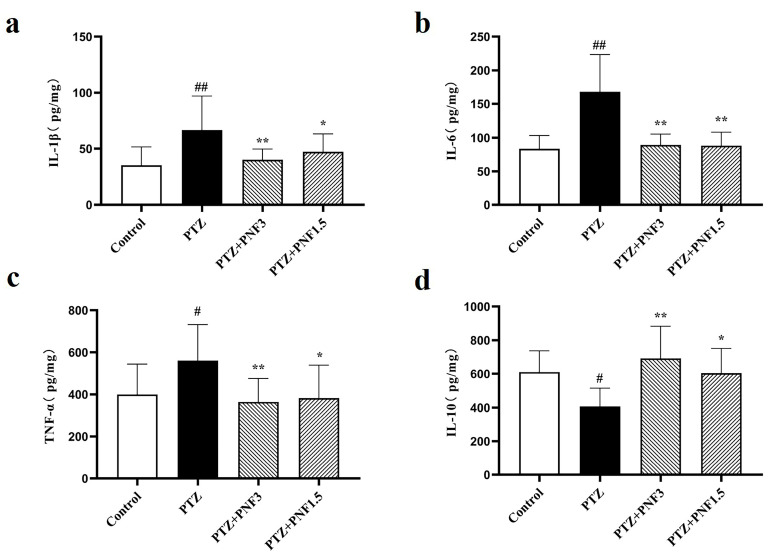
Effects of PNF on brain inflammatory cytokines in PTZ-kindled mice. (**a**) Levels of pro-inflammatory cytokines IL-1β; (**b**) levels of pro-inflammatory cytokines IL-6; (**c**) levels of pro-inflammatory cytokines TNF-α; (**d**) levels of anti-inflammatory cytokines IL-10. PNF treatment (1.5 g/kg and 3.0 g/kg) significantly reduced pro-inflammatory cytokines and restored IL-10 levels. The cytokines were detected with ELISA. A standard curve based on five standard concentrations was fitted using linear regression, and the model was regarded as statistically reliable when the coefficient of determination (R^2^) was greater than 0.99. The data are presented as mean ± SEM (*n* = 8); ^#^ *p* < 0.05, ^##^ *p* < 0.01 vs. control group; * *p* < 0.05, ** *p* < 0.01 vs. PTZ group.

Neuroinflammation is always accompanied by neurological diseases. To monitor the inflammation in the brain, cytokines were analyzed with ELISA after 30 days of treatment. The results showed that, compared with the control group, mice in the PTZ group exhibited significantly elevated levels of the pro-inflammatory cytokines IL-1β (Figure 4a), IL-6 (Figure 4b), and TNF-α (Figure 4c) in brain tissue (*p* < 0.05), while the level of the anti-inflammatory cytokine IL-10 (Figure 4d) was significantly decreased (*p* < 0.05). These findings indicate that chronic PTZ kindling induced a pronounced neuroinflammatory response in mice. Administration of PNF at 1.5 g/kg and 3.0 g/kg significantly reduced brain levels of IL-1β (Figure 4a), IL-6 (Figure 4b), and TNF-α (Figure 4c), while increasing IL-10 (Figure 4d) levels compared with the untreated PTZ group. These results suggest that PNF treatment attenuated PTZ-induced neuroinflammation in mice.

### 3.5. Effects of PNF on Hippocampal Glutamate in PTZ-Kindled Mice

To better understand the excitatory state of the epileptic brain, in vivo microdialysis was performed in conscious mice. The hippocampus was selected as the dialysis site, and glutamate (Glu), the most abundant excitatory neurotransmitter in the brain and a key mediator in epilepsy, was measured. The concentration of Glu in dialysates was determined using LC-MS/MS to assess changes in excitatory neurotransmitter levels in brain tissue.

**Figure 5:**
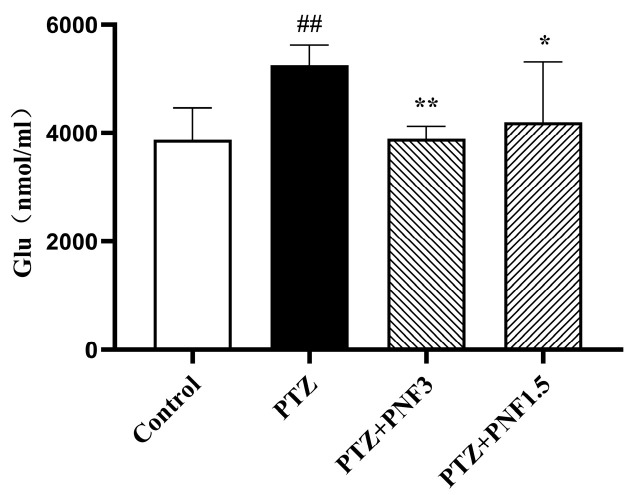
PNF reduces hippocampal glutamate levels in PTZ-kindled mice. In vivo hippocampal microdialysis followed by LC–MS/MS analysis revealed significantly elevated extracellular glutamate levels in PTZ-kindled mice. PNF treatment at both 1.5 g/kg and 3.0 g/kg markedly reduced glutamate concentrations. Data are presented as mean ± SEM (*n* = 4); ^##^ *p* < 0.01 vs. control group; * *p* < 0.05, ** *p* < 0.01 vs. PTZ group.

The results showed that (Figure 5), compared with the control group, hippocampal Glu levels were significantly elevated in PTZ-kindled mice (*p* < 0.01). Treatment with PNF, at both 3.0 g/kg and 1.5 g/kg, markedly reduced hippocampal Glu levels compared with the untreated PTZ group (*p* < 0.01 and *p* < 0.05, respectively). These findings indicate that PNF partially ameliorated PTZ-induced glutamatergic dysregulation in the hippocampus of chronically kindled mice.

### 3.6. Effects of PNF on Glutamate Modulating Related Proteins in the Brains of PTZ-Kindled Mice

The excitatory neurotransmitter glutamate is rapidly re-uptaken within milliseconds once released into the synaptic cleft. This process relies critically on glutamate transporters located on the membranes of astrocytes and neurons, particularly GLT-1 and GLAST, with astrocytic GLT-1 playing the predominant role. In astrocytes, glutamate is further converted to glutamine via GS, forming the Glu-Gln cycle.

**Figure 6:**
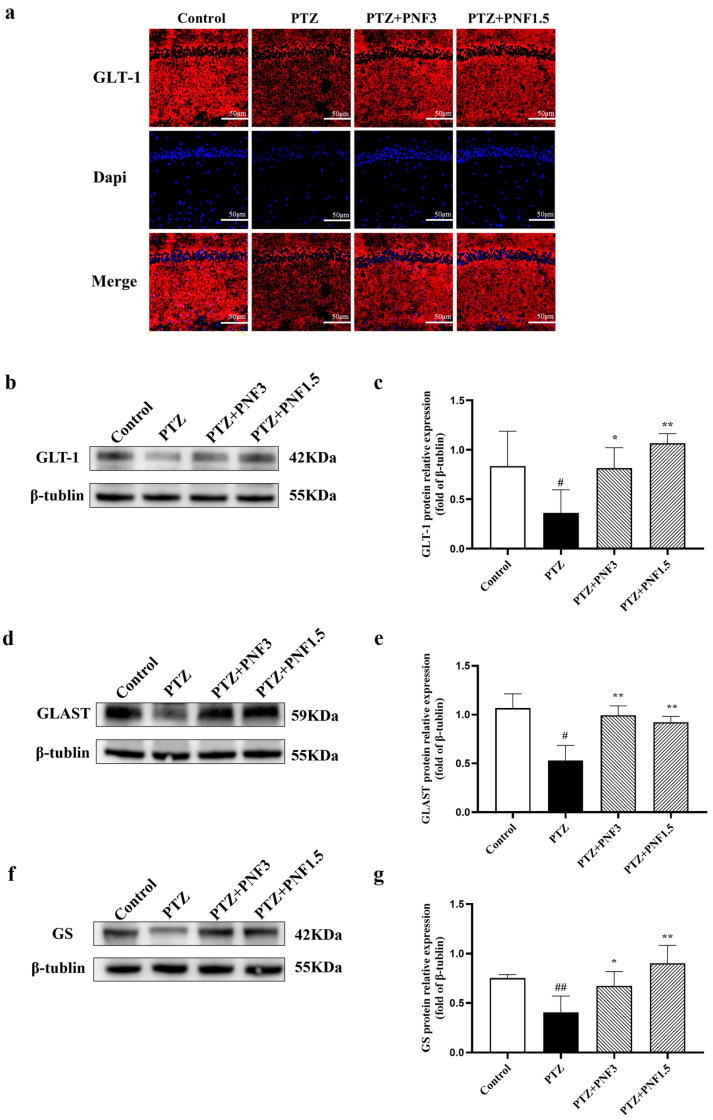
PNF restores expression of glutamate-modulating proteins in PTZ-kindled mice. (**a**) Representative immunofluorescence images showing GLT-1 expression in the hippocampus. GLT-1 fluorescence intensity was reduced in the PTZ group and increased following PNF treatment. Scale bar = 50 µm; (**b**) GLT-1 protein level; (**c**) Western blot analysis of GLT-1; (**d**) GLAST protein level; (**e**) Western blot analysis of GLAST; (**f**) glutamine synthetase (GS) protein level; (**g**) Western blot analysis of GS. Expression of all three proteins was significantly downregulated in the PTZ group and restored by PNF treatment. Data are presented as mean ± SEM (*n* = 3); ^#^ *p* < 0.05, ^##^ *p* < 0.01 vs. control group; * *p* < 0.05, ** *p* < 0.01 vs. PTZ group.

In this study, immunofluorescence staining was used to visualize GLT-1 in mouse brain tissue, and Western blotting was performed to measure protein expression of GLT-1, GLAST, and GS. Immunofluorescence results revealed that GLT-1 intensity in the PTZ group were obviously weaker than those in the control group (Figure 6a). Consistently, Western blot analysis demonstrated that GLT-1 protein levels in the PTZ group were significantly reduced (Figure 6b,c), accompanied by downregulation of GLAST and GS (Figure 6d–g).

Following PNF treatment, GLT-1 immunofluorescence intensity in the brain was visibly enhanced (Figure 6a), and Western blot analysis confirmed a significant increase in GLT-1 protein expression (Figure 6b,c). Moreover, GLAST and GS protein levels were also markedly higher in PNF-treated mice compared with the untreated PTZ group (Figure 6d–g).

## 4. Discussion

In this study, it has been demonstrated that Panax notoginseng flower (PNF) exerts distinct neuroprotective and antiepileptic effects in a chronic PTZ-kindled mouse model. These preliminary findings indicate that PNF could regulate glutamate homeostasis, suppresses systemic and cerebral inflammation, promote the neuronal recovery, and mitigates seizure severity.

Epilepsy is a chronic neurological disorder characterized by recurrent seizures and neuronal hyperexcitability. A significant proportion of patients remain refractory to current antiseizure medications, highlighting the urgent need for novel, effective, and safe therapeutic strategies [3]. In this context, traditional medicinal plants, especially species of the Panax genus, have gained attention due to their multi-targeted pharmacological effects. While ginsenosides from Panax ginseng and Panax notoginseng roots have been studied in epilepsy and other CNS disorders, the Panax notoginseng flower (PNF) remains relatively underexplored. The present study addresses this gap and demonstrates that PNF extract exhibits antiepileptic and neuroprotective effects in a PTZ-induced chronic epilepsy model by modulating glutamate homeostasis and inflammatory pathways.

Compared to the well-characterized roots, Panax notoginseng flowers contain a richer concentration of protopanaxadiol-type saponins, particularly ginsenoside Rb1, Rc, and Rd [13,14]. Our study is among the first to investigate the pharmacological activity of this botanical part in an epilepsy context. This novel application distinguishes the current research from earlier studies focusing on root extracts or isolated ginsenosides. Importantly, PNF was administered as a full-spectrum extract, supporting its potential use in real-world traditional medicine formulations.

One of the most compelling findings is the ability of PNF to restore glutamate homeostasis in the hippocampus. Dysregulation of glutamate clearance and metabolism is a central mechanism in seizure pathophysiology. Under normal conditions, astrocytic glutamate transporters GLT-1 and GLAST, and the enzyme glutamine synthetase (GS), regulate the glutamate-glutamine cycle and prevent excitotoxicity [7,17,18]. PTZ-induced chronic epilepsy disrupted these homeostatic mechanisms, leading to elevated extracellular glutamate levels and neuronal injury. Consistent with prior reports [9], the current study observed significant reductions in GLT-1, GLAST, and GS expression in PTZ-kindled mice, along with increased glutamate levels measured via LC-MS/MS microdialysis. PNF treatment restored the expression of these proteins and reduced extracellular glutamate, indicating enhanced glutamate uptake and metabolic conversion. This mechanism is likely mediated through ginsenoside Rb1, which has previously been shown to activate GPCR-Gs signaling and upregulate astrocytic function [11]. These results strengthen the rationale for targeting astrocyte-mediated glutamate regulation in epilepsy and position PNF as a promising agent with multimodal effects.

Chronic neuroinflammation contributes to epileptogenesis, seizure recurrence, and neuronal damage [19]. In the PTZ-kindled model, upregulation of pro-inflammatory cytokines (IL-1β, IL-6, and TNF-α) and downregulation of IL-10 were observed in both serum and brain tissue [20,21,22]. PNF significantly reversed this pro-inflammatory profile, indicating systemic and central anti-inflammatory effects. This dual action suggests that PNF’s bioactive components likely cross the blood–brain barrier and exert immunomodulatory effects at multiple levels. These results are supported by prior studies demonstrating anti-inflammatory effects of ginsenosides in other models of neuroinflammation [23]. Moreover, other anti-inflammatory compounds such as myricetin, piroxicam, and dexamethasone have been shown to attenuate seizures and hippocampal injury in PTZ-kindled mice [24,25,26]. This finding positions PNF as a potential candidate in adjunctive epilepsy therapy that combines anti-inflammatory and neurochemical modulation.

Histological analysis revealed significant neuronal loss in PTZ-kindled mice, especially in the hippocampal CA1 region and cerebral cortex. These observations are in agreement with previous studies showing seizure-induced neuronal degeneration [27]. PNF treatment preserved NeuN-positive neuronal populations in these regions, which may reflect both its glutamate-modulating and anti-inflammatory effects.

Although this study did not include cognitive testing, preservation of hippocampal neurons implies that PNF may also have beneficial effects on learning, memory, and mood comorbidities commonly associated with chronic epilepsy.

Most epilepsy studies on Panax species have focused on Panax ginseng root or total ginsenosides. For instance, intranasal Rb1 administration was shown to reduce seizure activity in PTZ models [16]. However, these approaches often require chemical purification or invasive administration routes. Our study is the first to demonstrate that orally administered full-spectrum PNF extract can produce comparable, if not superior, antiepileptic effects. This finding underscores the translational relevance of PNF as a practical and culturally acceptable therapeutic agent with a history of traditional use in Chinese medicine.

Although the current findings are promising, several limitations should be acknowledged. First, the active constituents of PNF responsible for the observed effects were not isolated. Future studies using fractionation and pharmacokinetic profiling will help identify the bioactive components. Second, the model used focused on chemically induced seizures and may not fully replicate genetic or structural causes of epilepsy. Third, while transporter and cytokine expression was quantified, additional mechanistic pathways (e.g., oxidative stress, mitochondrial dysfunction) were not explored. Behavioral assessments and EEG monitoring in future studies could also provide further insight into the functional consequences of PNF treatment.

In conclusion, Panax notoginseng flower (PNF) extract confers significant neuroprotective and antiepileptic effects in PTZ-kindled mice through restoration of glutamate homeostasis, upregulation of astrocytic transporters, suppression of systemic and cerebral inflammation. These findings support the therapeutic potential of PNF as a complementary and multifunctional approach for epilepsy management. Further research is needed to elucidate its full pharmacological profile and facilitate clinical translation.

## Data Availability

The raw data supporting the conclusions of this article will be made available by the authors on request.
